# Intrahippocampal glucocorticoids generated by 11β-HSD1 affect memory in aged mice

**DOI:** 10.1016/j.neurobiolaging.2014.07.007

**Published:** 2015-01

**Authors:** Joyce L.W. Yau, Nicola Wheelan, June Noble, Brian R. Walker, Scott P. Webster, Christopher J. Kenyon, Mike Ludwig, Jonathan R. Seckl

**Affiliations:** a Centre for Cognitive Aging and Cognitive Epidemiology, University of Edinburgh, Edinburgh, UK; b Endocrinology Unit, British Heart Foundation Centre for Cardiovascular Science, The Queen's Medical Research Institute, University of Edinburgh, Edinburgh, UK; c Centre for Integrative Physiology, University of Edinburgh, Edinburgh, UK

**Keywords:** Aging, Stress, Corticosterone, Spatial memory, 11β-HSD1, Hippocampus

## Abstract

11Beta-hydroxysteroid dehydrogenase type 1 (11β-HSD1) locally amplifies active glucocorticoids within specific tissues including in brain. In the hippocampus, 11β-HSD1 messenger RNA increases with aging. Here, we report significantly greater increases in intrahippocampal corticosterone (CORT) levels in aged wild-type (WT) mice during the acquisition and retrieval trials in a Y-maze than age-matched 11β-HSD1^−/−^ mice, corresponding to impaired and intact spatial memory, respectively. Acute stress applied to young WT mice led to increases in intrahippocampal CORT levels similar to the effects of aging and impaired retrieval of spatial memory. 11β-HSD1^−/−^ mice resisted the stress-induced memory impairment. Pharmacologic inhibition of 11β-HSD1 abolished increases in intrahippocampal CORT levels during the Y-maze trials and prevented spatial memory impairments in aged WT mice. These data provide the first in vivo evidence that dynamic increases in hippocampal 11β-HSD1 regenerated CORT levels during learning and retrieval play a key role in age- and stress-associated impairments of spatial memory.

## Highlights

• We followed intrahippocampal corticosterone (CORT) levels in mice during memory testing in a Y-maze.
• Aged 11beta-hydroxysteroid dehydrogenase type 1 (11β-HSD1^−/−^) mice resists age-related spatial memory decline in the Y-maze.
• A lower dynamic rise in intrahippocampal CORT levels associates with better memory.
• Acute stress increases intrahippocampal CORT and impairs memory in young mice.
• 11β-HSD1 inhibition reduces intrahippocampal CORT and improves memory in aged mice.

## 1 Introduction

Both aging and stress induce memory impairments notably in hippocampus-associated cognitive functions. Stress activates the hypothalamic-pituitary-adrenal (HPA) axis causing acute elevation of blood glucocorticoid levels, whereas in a subset of aged individuals, plasma glucocorticoid levels are elevated, and this correlates with hippocampal-associated memory deficits (Lupien et al., 1998).

Glucocorticoid effects on memory are mediated via mineralocorticoid receptors (MRs) and glucocorticoid receptors (GRs). Both are highly expressed in the hippocampus, a structure involved in the formation of new memories and in the negative feedback control of the HPA axis (Eichenbaum et al., 2007, Tasker and Herman, 2011). Glucocorticoid action within specific cells is modulated by 11beta-hydroxysteroid dehydrogenase type 1 (11β-HSD1), which catalyzes the local regeneration of active cortisol and corticosterone (CORT) from inert 11-keto derivatives (cortisone in humans, 11-dehydrocorticosterone in rodents) (Holmes and Seckl, 2006). 11β-HSD1 is highly expressed in regions of the adult brain that underpin memory including the hippocampus and cortex (Moisan et al., 1990, Sandeep et al., 2004).

Independent of circulating glucocorticoids, the local regeneration of active steroids by 11β-HSD1 influences memory deficits with aging (Yau and Seckl, 2012). Thus, 11β-HSD1 expression in mouse hippocampus and cortex increases with aging, correlating with impaired spatial memory. Also forebrain-specific overexpression of 11β-HSD1 in mice accelerates age-dependent cognitive loss (Holmes et al., 2010). Conversely, mice genetically deficient in 11β-HSD1 resist age-dependent cognitive decline (Yau et al., 2001, Yau et al., 2007), and strikingly, short-term pharmacologic inhibition of 11β-HSD1 reverses memory deficits in aged mice (Sooy et al., 2010). Moreover, short-term treatment with a nonselective 11β-HSD inhibitor, carbenoxolone, improved aspects of memory in a small cohort of elderly humans and subjects with type 2 diabetes (Sandeep et al., 2004). Lower hippocampal tissue CORT levels, as measured ex vivo in aged 11β-HSD1 knockout mice, have been invoked to explain the cognitive protection (Yau et al., 2007). However, a direct causal effect of 11β-HSD1–amplified hippocampal CORT levels on impairment of spatial memory has not been shown in vivo.

Prolonged exposure to stress and high CORT levels have been shown to impair memory in rodents (Bodnoff et al., 1995, Dachir et al., 1993, Luine et al., 1994). Evidence that glucocorticoids mediate the effects of stress and aging on memory largely derive from measurements of single plasma samples taken at a time point dissociated from the learning task. Circulating glucocorticoids are, however, mostly bound to corticosteroid-binding globulin, and only free plasma glucocorticoids (∼5%) cross the blood-brain barrier. Brain extracellular fluid glucocorticoid concentrations reflect this free fraction and are, thus, a better indicator of the hormone available for binding to intracellular MR and GR than plasma levels. Moreover, glucocorticoids measured in extracellular fluid also take account of local brain 11β-HSD1 activity that could fluctuate as a function of substrate availability, independent of the degree of HPA activation.

In the present study, we used in vivo microdialysis in freely behaving mice to examine (1) the effects of aging and/or stress on intrahippocampal CORT levels and memory during learning and retrieval; and (2) the role of 11β-HSD1 in these processes.

## 2 Methods

### 2.1 Animals

Male mice homozygous for a targeted disruption of *hsd11b1*, congenic on the C57BL/6J genetic background, and wild-type (WT) C57BL/6J mice were bred in-house under standard conditions (12 hours light-dark cycle) with food and water ad libitum until experimentation at either 6–8 months old (young) or 24–26 months old (aged) (Carter et al., 2009, Kotelevtsev et al., 1997). All animal procedures were approved by the local ethical committee of University of Edinburgh and carried out in strict accordance with the UK Animals (Scientific Procedures) Act, 1986.

### 2.2 Y-maze task

A 2-trial Y-maze task was chosen to assess hippocampal-dependent spatial recognition memory (Conrad et al., 1996, Yau et al., 2011). The size of the maze allowed for in vivo microdialysis in freely behaving mice. Each mouse was placed at the end of the start arm and allowed to explore the maze with 1 arm blocked (novel arm) for 5 minutes (trial 1). After an intertrial interval (ITI) of 2 hours, the mouse was returned to the maze and allowed to explore all the 3 arms for 5 minutes (trial 2) (Yau et al., 2011). The time spent in the novel arm was calculated with ANY-maze software (Stoelting, Dublin, Ireland). All behavioral testing was carried out in the morning (9:00–12:00 AM).

### 2.3 Surgery

Mice were anesthetized with isoflurane (2.5%) and fixed in a stereotaxic frame using a tooth bar with the attached mouse anesthetic mask (David Kopf Instruments, Tujunga, CA, USA). A microdialysis guide cannula (CMA/7; CMA Microdialysis, Kista, Sweden) was implanted at a position just entering the dorsal hippocampus, a site shown to be important for spatial learning (Moser et al., 1995) (coordinates relative to bregma: anteroposterior − 2.3 mm, lateral +1.5 mm [midline], dorsoventral − 1.5 mm from the skull surface). Dental cement and 2 small anchor screws secured the guide cannula and a small metal peg (for later connection to a liquid swivel) on the skull. Mice were allowed to recover for 4 days before the microdialysis procedure.

### 2.4 Microdialysis

Under isoflurane (2.5%) anesthesia, a dialysis probe (CMA/7; CMA Microdialysis, length 1 mm, molecular cutoff 6 kDa, and membrane outer diameter 0.24 mm) was introduced via the preimplanted guide cannula to protrude 1 mm into the hippocampus. Mice were individually housed in a clear round-bottom bowl system for freely moving animals (CMA/120; CMA Microdialysis) with free access to water and food. The probe was perfused continuously overnight with sterile artificial cerebrospinal fluid at 0.3 μL per minute using a microinfusion pump to allow extracellular metabolites to equilibrate with the dialysate and to allow the animal to acclimatize to the liquid swivel counterbalance arm system. Fluorethylenepolymer tubing with a dead volume of 1.2 μL per 100 mm length was used for collection of microdialysis samples from the probe into cooled plastic vials using an automated refrigerated fraction collector (CMA 470; CMA Microdialysis).

### 2.5 Histology

At the end of each microdialysis experiment, the mice were culled by cervical dislocation and brains removed and frozen on powdered dry ice. Histologic verification of probe placement was determined from 30 μm cryostat sections stained with pyronin. Only data from mice with the microdialysis probe placed correctly in the hippocampus (>90% of mice) were analyzed.

### 2.6 11β-HSD1 inhibition

The novel compound UE2316 ([4-(2-chlorophenyl-4-fluoro-1-piperidinyl][5-(1H-pyrazol-4-yl)-3-thienyl]-methanone) was synthesized by High Force Ltd, UK, according to methods previously described (Webster et al., 2011). In vitro screening of UE2316 potency in HEK293 cells stably transfected with hsd11b1 showed a lower median inhibitory concentration (IC_50_) (human 42 nM, rat 80 nM, mouse 162 nM) than our previously reported compound UE1961 (Sooy et al., 2010). UE2316 showed selective inhibition of 11β-HSD1 with no significant off-target activities in selectivity screening, including against 11β-HSD2 (IC_50_ > 10 μM), 17β-HSD1 (IC_50_ > 10 μM), and GR (*K*_*d*_ > 10 μM).

### 2.7 11β-HSD1 activity assays

Brain samples (hippocampus and cortex) were homogenized and assayed for 11-ketosteroid reductase activity as previously described (Sooy et al., 2010) and were expressed as the percentage conversion of [^3^H]-11-dehydrocorticosterone to [^3^H]-CORT.

### 2.8 CORT assays

Total CORT levels in plasma were measured using an in-house radioimmunoassay (RIA) with [3H]-CORT (Yau et al., 2011). For ex vivo hippocampal CORT levels, steroids were extracted by solvolysis from dissected tissues before RIA (Yau et al., 2011). Intrahippocampal CORT levels were measured in 10 μL dialysate samples using an RIA with [^125^Iodine]-CORT because of the greater sensitivity required to detect the much lower brain CORT levels. The intra-assay coefficient of variation was 4% and detection limit of 0.0014 pmol.

### 2.9 Experimental design

#### 2.9.1 Study 1: intrahippocampal CORT levels during simultaneous Y-maze testing in WT and 11β-HSD1^−/−^ mice

Young and aged mice of each genotype underwent surgery and implantation of a microdialysis probe into the dorsal hippocampus as described previously. After overnight perfusion, the flow rate was increased to 1 μL/minute and dialysate samples were collected every 10 minutes during the spatial memory task. Fluorethylenepolymer tubing from the probe outlet was threaded through an assembly of interconnected wires and linked via the metal peg to a liquid swivel assembly that allowed unrestricted movement of the mouse in the Y-maze. After 1 hour of baseline sampling, the mouse was placed in the start arm of the maze for trial 1 and returned to the containment bowl during the 2-hour ITI before trial 2 in the maze. Finally, they were returned to their containment bowls for a further hour of sampling at the end of maze testing. Microdialysis samples were stored at − 80 °C for later determination of CORT concentrations.

#### 2.9.2 Study 2: effect of acute stress during Y-maze testing on spatial memory

Tail nick was the chosen acute stressor because the blood sample obtained within 2 minutes of venesection allows for plasma CORT measurements and because it also provokes a subsequent pituitary-driven CORT increase (Vahl et al., 2005) to compromise memory. Two days before Y-maze testing, tail nick blood (∼30 μL) was sampled in the morning (08:00–09:00 AM) from control and 11β-HSD1^−/−^ mice for basal CORT levels. During Y-maze testing, tail nicks were administered immediately after trial 1 (acquisition) and just before trial 2 (retrieval) to examine the effects of stress on spatial memory in young and aged mice (Fig. 2A). The following morning, mice were culled by cervical dislocation and brains removed, dissected, snap frozen on powdered dry ice, and stored at − 80 °C for later analysis of tissue CORT levels.

#### 2.9.3 Study 3: effect of acute stress on intrahippocampal CORT levels during Y-maze testing

The procedure used for study 1 was applied to young and aged mice of each genotype with an additional tail nick stress administered before retrieval (trial 2).

#### 2.9.4 Study 4: effect of 11β-HSD1 inhibition on intrahippocampal CORT levels during Y-maze testing in aged mice

To avoid the stress of daily injections, UE2316 was administered by voluntary oral consumption. UE2316 (10 mg/kg, twice daily) or vehicle (2% dimethylsulfoxide) was administered in sucralose-sweetened gelatin with added red food dye and strawberry essence (Zhang, 2011) to singly housed mice. UE2316 was dissolved in dimethylsulfoxide and then incorporated into gelatin. Before the experiment, mice were trained to eat the vehicle jelly immediately on presentation in their cages. Initially, young WT controls (*n* = 5 per group) were treated for 4 days with drug or vehicle jelly to test for effective inhibition of brain 11β-HSD1. Mice were culled 1 hour after the last drug dose and brains dissected into regions and stored frozen for assay of 11β-HSD1 activity.

WT mice at 24 months were treated with UE2316 (10 mg/kg) or vehicle for 2 weeks to test for the effects on intrahippocampal CORT levels during spatial memory performance in the Y-maze. Treatment started before surgery and continued during microdialysis and Y-maze testing.

### 2.10 Statistical analysis

Data were analyzed using either a 1- or a 2-way analysis of variance followed by Scheffe post hoc test as appropriate for between-group comparisons. Microdialysis CORT data were analyzed by repeated-measures analysis of variance with genotype and time as the variables. The percent time spent in the novel arm versus the other arms of the Y-maze within each group was analyzed by Student paired *t* test. For each group, plasma CORT levels before and during the Y-maze were compared by Student paired *t* test. Significance was set at *p* < 0.05. All data were presented as mean ± standard error of the mean.

## 3 Results

### 3.1 Higher intrahippocampal CORT levels associate with impaired spatial memory retention during the Y-maze task in aged WT but not 11β-HSD1^−/−^ mice

**Fig. 1:**
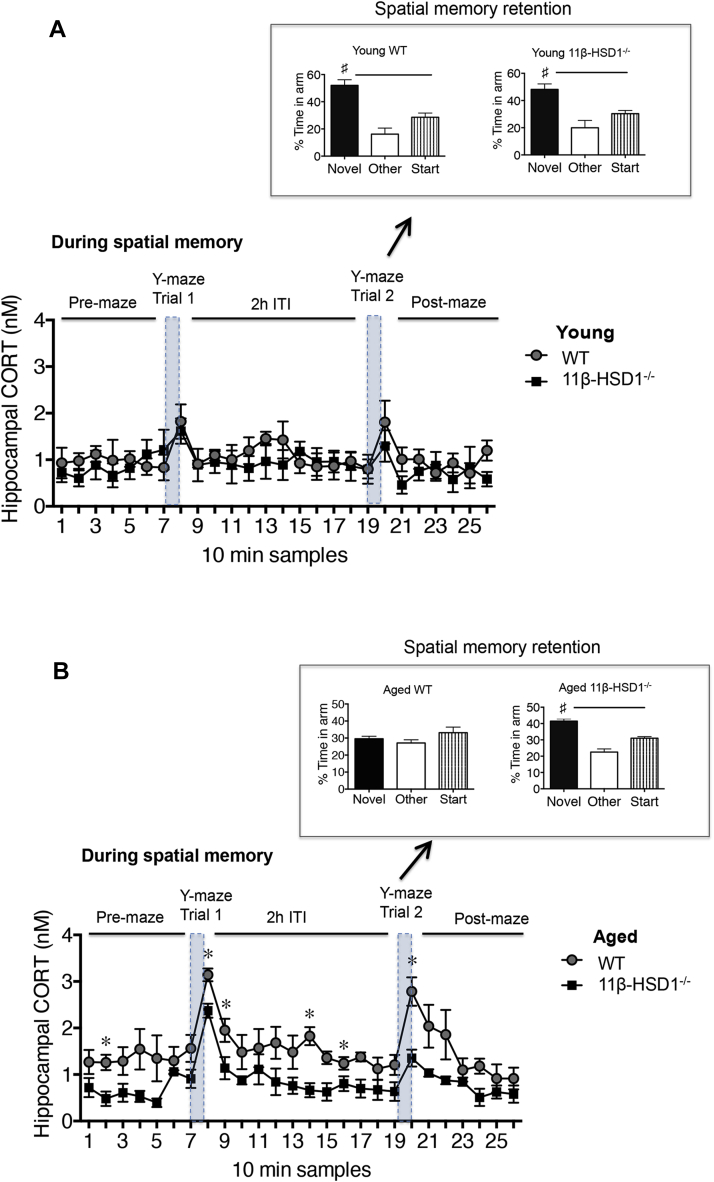
Effects of 11beta-hydroxysteroid dehydrogenase type 1 (11β-HSD1) deficiency and aging on intrahippocampal corticosterone (CORT) levels during spatial memory performance in a Y-maze. (A) Young 6-month-old wild-type (WT) and 11β-HSD1^−/−^ mice (*n* = 4–5 per genotype) and (B) aged 24-month-old WT and 11β-HSD1^−/−^ mice (*n* = 5 per genotype) showing intrahippocampal CORT levels measured throughout spatial memory performance (in consecutive 10-minute dialysate samples, * *p* < 0.05 vs. equivalent time point in 11β-HSD1^−/−^ mice) and Y-maze spatial memory retention during trial 2 after a 2-hour intertrial interval (ITI). Shaded areas indicate 5 minutes explorations of Y-maze in trials 1 (acquisition) and 2 (retrieval); # *p* < 0.05 versus start and other arms.

The Y-maze task caused small but similar elevations (*p* < 0.01) in intrahippocampal CORT levels (measured in dialysate samples taken every 10 minutes) during acquisition (trial 1) and retrieval (trial 2) in young WT and 11β-HSD1^−/−^ mice (Fig. 1A). Spatial memory of the maze over the 2-hour ITI was retained in both groups of mice with significantly more time spent exploring the novel arm than previously visited arms (*p* < 0.05, Fig. 1A).

Temporal increases in intrahippocampal CORT levels in aged WT mice during the Y-maze trials were greater than in young WT mice (*F*_1,181_ = 34, *p* < 0.0001, Fig. 1A and B) and also greater than aged 11β-HSD1^−/−^ mice (*F*_1,202_ = 79.9, *p* < 0.0001, Fig. 1B). Intrahippocampal CORT levels increases during Y-maze trials in aged 11β-HSD1^−/−^ mice were similar to those in young 11β-HSD1^−/−^ mice (*F*_1,191_ = 0.55, *p* = 0.89, Fig. 1A and B). Correspondingly, spatial memory was impaired in aged WT mice but remained intact in 11β-HSD1^−/−^ mice (Fig. 1B). The impaired spatial memory in aged WT mice was not because of vision or locomotion problems because when tested with a 1-minute rather than a 2-hour ITI, they could recall which arms they had already visited and exhibited neophilia (Supplementary Fig. S1).

### 3.2 Acute stress elevates plasma CORT levels and impairs spatial memory in WT but not 11β-HSD1^−/−^ mice

**Fig. 2:**
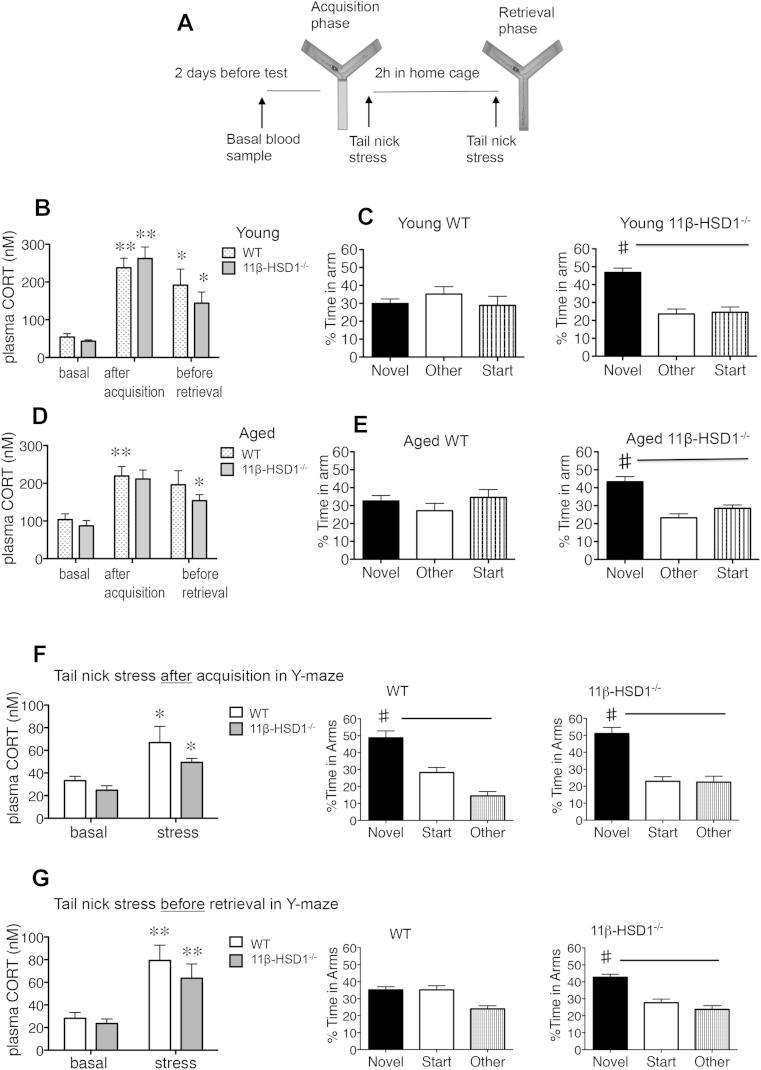
Acute stress elevates plasma corticosterone (CORT) levels and impairs spatial memory retrieval in wild-type (WT) but not 11beta-hydroxysteroid dehydrogenase type 1 (11β-HSD1^−/−^) mice. (A) Schematic diagram showing tail-nick blood-sampling stress during Y-maze performance. (B) Plasma CORT levels in young 6-month-old WT and 11β-HSD1^−/−^ mice (*n* = 9 per genotype) during Y-maze testing (** *p* < 0.001, * *p* < 0.05 vs. basal CORT) and (C) associated impaired and intact Y-maze spatial memory retention during trial 2 in young WT and 11β-HSD1^−/−^ mice, respectively (# *p* < 0.05 vs. start and other arms). (D) Plasma CORT levels in aged 24-month-old WT and 11β-HSD1^−/−^ mice (*n* = 10–11 per genotype) during Y-maze testing (** *p* < 0.001, * *p* < 0.05 vs. basal CORT) and (E) associated impaired and intact spatial memory retention during Y-maze trial 2 in aged WT and 11β-HSD1^−/−^ mice, respectively (# *p* < 0.05 vs. start and other arms). (F) Effects of tail-nick stress applied to young 8-month-old WT and 11β-HSD1^−/−^ mice immediately after acquisition (trial 1, *n* = 7 per genotype) or (G) immediately before retrieval (trial 2, *n* = 10 per genotype), on plasma CORT levels and corresponding Y-maze spatial memory retention after a 2-hour intertrial interval; * *p* < 0.05 versus corresponding basal levels, paired *t* test, and # *p* < 0.05 versus start and other arms.

Tail nick stress applied to young WT mice during the Y-maze task immediately after acquisition (trial 1) and just before retrieval (trial 2) (Fig. 2A) caused marked 5- to 6-fold elevations of plasma CORT levels compared with basal CORT levels in samples collected from the same mice 2 days earlier (*F*_2,47_ = 33.47, *p* < 0.0001, Fig. 2B). These young stressed WT mice now showed impaired spatial memory like aged WT controls, spending similar amounts of time exploring the 3 arms (Fig. 2C). In contrast, young 11β-HSD1^−/−^ mice resisted the stress-induced memory impairment and spent more time in the novel arm than previously visited arms (paired *t* test, *p* < 0.01); time in novel arm was more than in young WT controls (*F*_2,48_ = 6.21, *p* < 0.01, Fig. 2C).

Basal plasma CORT levels increased with aging in both genotypes (*p* < 0.05, Fig. 2B and D). Tail nick stress applied during Y-maze testing further increased circulating CORT levels in aged mice (*F*_2,57_ = 14.02, *p* < 0.001) with no effect of genotype (*F*_1,57_ = 0.84, *p* = 0.36) (Fig. 2D). Stressed and aged WT mice were impaired in the Y-maze (Fig. 2E). In contrast, aged 11β-HSD1^−/−^ mice resisted the combined memory impairing effects of age and stress and showed intact spatial memory (Fig. 2E).

Both genotype and age influenced hippocampal tissue CORT levels measured ex vivo after behavioral testing (genotype: *F*_1,34_ = 10.6, *p* < 0.01 and age: *F*_1,34_ = 4.3, *p* < 0.05, Supplementary Table S1). CORT was 32% lower in aged 11β-HSD1^−/−^ mice than matched WT controls (*F*_1,19_ = 9.3, *p* < 0.01) but was not different from young 11β-HSD1^−/−^ mice (Table S1). CORT concentrations increased with age in WT controls (37% higher than young, *F*_1,16_ = 6.4, *p* < 0.05) but not in 11β-HSD1^−/−^ mice, despite both genotypes showing similar increases in basal plasma CORT levels (Table S1).

To determine which of the 2 tail-nick stress applications during the Y-maze impaired spatial memory in young WT mice, we compared responses with each tail-nick stress in separate groups of young mice. Tail nicks administered immediately after trial 1 (acquisition) caused similar increases in plasma CORT levels in WT and 11β-HSD1^−/−^ mice (*F*_1,24_ = 13.6, *p* < 0.01, Fig. 2F) and did not impair spatial memory. Tail nicks before trial 2 (retrieval) increased plasma CORT levels to a similar extent (*F*_1,32_ = 16.9, *p* < 0.001) but only WT mice showed spatial memory impairment (Fig. 2G). These results indicate that previous stress compromises memory retrieval by a process that is independent of plasma CORT changes.

### 3.3 Acute stress elevates intrahippocampal CORT levels and impairs retrieval of spatial memory in WT but not 11β-HSD1^−/−^ mice

**Fig. 3:**
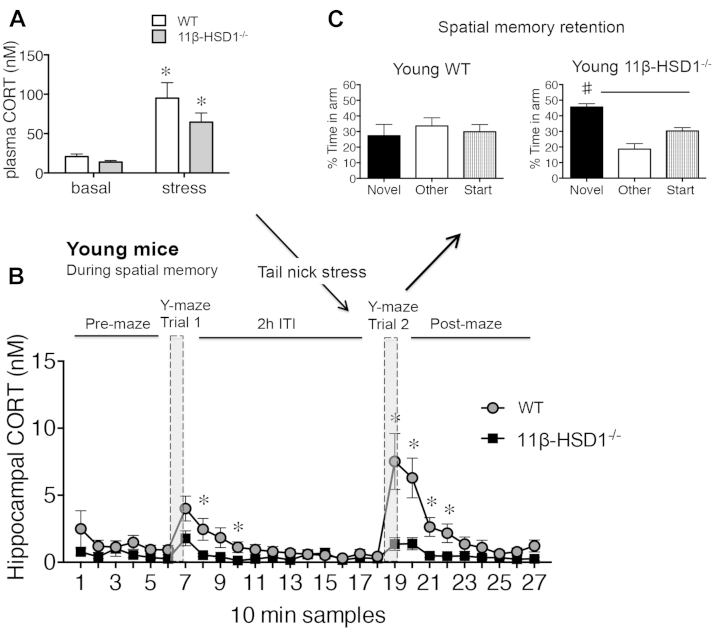
Acute stress increases intrahippocampal corticosterone (CORT) levels and impairs spatial memory retrieval in young wild-type (WT) but not 11beta-hydroxysteroid dehydrogenase type 1 (11β-HSD1^−/−^) mice. (A) Plasma CORT levels after tail-nick stress and its effect on intrahippocampal CORT dynamics (in consecutive 10-minute dialysate samples, * *p* < 0.05 vs. equivalent time point in 11β-HSD1^−/−^ mice) during Y-maze performance (B) and resulting spatial memory retention after a 2-hour intertrial interval (ITI) (# *p* < 0.05 vs. start and other arms) (C) in young 6-month-old WT (*n* = 8) and 11β-HSD1^−/−^ mice (*n* = 7).

The fact that memory retrieval was unimpaired in 11β-HSD1^−/−^ mice despite plasma CORT levels being elevated to a similar extent as WT controls suggests that local CORT regeneration could play an important role. To test this hypothesis, we assessed local hippocampal CORT levels in vivo by microdialysis during the Y-maze behavioral tests. Tail-nick stress just before trial 2 in the Y-maze not only increased plasma CORT levels in young WT mice (Fig. 3A) but also increased intrahippocampal CORT to levels that were even higher than those caused by trial 1 (i.e., without tail-nick stress) (*F*_1,96_ = 8.1, *p* < 0.01, Fig. 3B) and caused spatial memory impairment (Fig. 3C). Stress-induced increases in intrahippocampal CORT in young WT mice in trial 2 were comparable with those of aged WT mice during Y-maze training alone (i.e., without the additional tail nick stress) (Supplementary Fig. S2).

Stress-induced increases in plasma CORT in young 11β-HSD1^−/−^ mice were similar to those of WT controls (Fig. 3A). However, during trial 2 in the Y-maze, temporal increases in intrahippocampal CORT levels (*F*_1,89_ = 27.1, *p* < 0.0001, Fig. 3B) were lower than WT controls and spatial memory appeared to be unimpaired (i.e., time spent in novel was greater than in previously visited arms, *p* < 0.05, Fig. 3C).

**Fig. 4:**
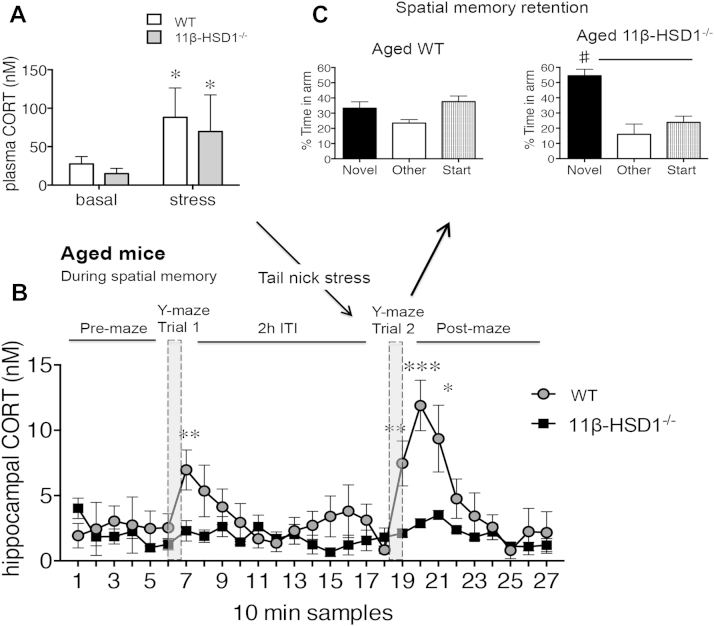
Aged 11beta-hydroxysteroid dehydrogenase type 1 (11β-HSD1^−/−^) mice maintain low intrahippocampal corticosterone (CORT) levels and intact spatial memory despite tail-nick stress before retrieval. (A) Plasma CORT levels after tail-nick stress and its effect on intrahippocampal CORT dynamics (in consecutive 10-minute dialysate samples, *** *p* < 0.001, ** *p* < 0.01, * *p* < 0.05 vs. equivalent time point in 11β-HSD1^−/−^ mice) during Y-maze performance (B) and resulting spatial memory retention after a 2-hour intertrial interval (ITI) (# *p* < 0.05 vs. start and other arms) (C) in aged 24- to 26-month-old wild-type (WT) (*n* = 5) and 11β-HSD1^−/−^ mice (*n* = 6).

In aged mice, tail-nick stress before trial 2 increased plasma CORT levels in both WT and 11β-HSD1^−/−^ mice (*p* < 0.05, Fig. 4A), but the temporal rise in intrahippocampal CORT levels during trial 2 was greater than trial 1 for WT mice (*F*_1,46_ = 6.2, *p* < 0.05, Fig. 4B) but not for 11β-HSD1^−/−^ mice. Significantly, the enhanced intrahippocampal CORT response in aged WT mice was associated with impaired spatial memory that was not observed in aged 11β-HSD1^−/−^ mice with attenuated intrahippocampal CORT response (Fig. 4C).

### 3.4 Pharmacologic inhibition of 11β-HSD1 reduces intrahippocampal CORT levels in aged mice and improves spatial memory

**Fig. 5:**
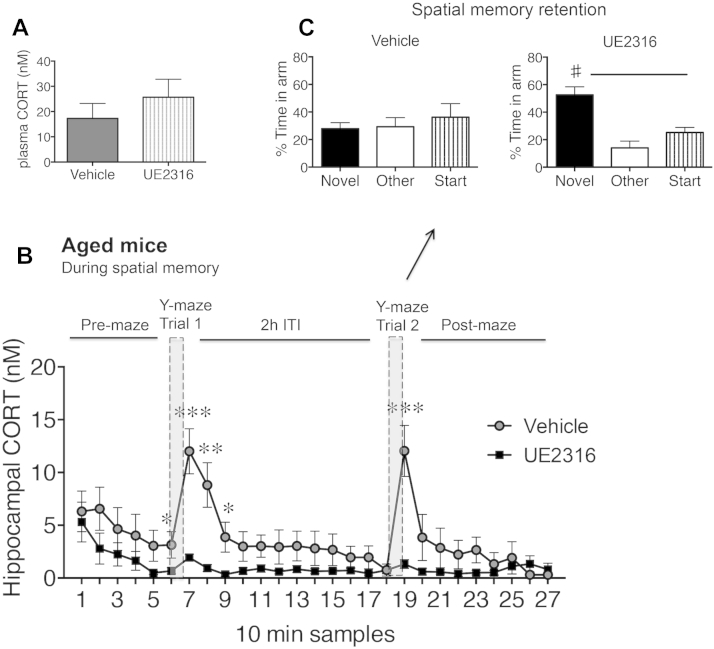
UE2316 treatment reduces intrahippocampal corticosterone (CORT) levels during Y-maze learning and improves spatial memory in aged wild-type (WT) mice. (A) Morning basal plasma CORT levels in UE2316 and vehicle-treated 24-month-old WT mice (B) intrahippocampal CORT dynamics during Y-maze learning after 2 weeks of UE2316 (10 mg/kg twice daily or vehicle orally in jelly, *n* = 5–7 per group). *** *p* < 0.001, ** *p* < 0.01, * *p* < 0.05 compared with corresponding time point of UE2316 treated group and (C) Y-maze spatial memory retention (# *p* < 0.05 vs. start and other arms) after a 2-hour intertrial interval (ITI).

Aged WT mice treated orally with UE2316 (10 mg/kg, twice daily in jelly, a dose shown to effectively inhibit brain 11β-HSD1, Supplementary Fig. S3) for 2 weeks showed no effect on plasma CORT levels (Fig. 5A) but blocked the enhancement of temporal and dynamic increases in intrahippocampal CORT levels normally seen in vehicle-treated controls (*F*_1,269_ = 85.7, *p* < 0.0001, Fig. 5B). This reduced response coincided with improved spatial memory (time in novel arm with UE2316 was greater than with vehicle, *p* < 0.05, Fig. 5C), whereas vehicle-treated aged controls were impaired (Fig. 5C).

## 4 Discussion

The present study highlights a direct role of 11β-HSD1 in age- and stress-associated impairment of spatial memory. We provide evidence that in aged mice and in stressed young mice, a component part of the elevations in intrahippocampal CORT levels during Y-maze testing is because of local 11β-HSD1 activity and that this 11β-HSD1 component can cause impaired spatial memory. Importantly, 11β-HSD1^−/−^ mice, that cannot reactivate hippocampal CORT and, hence, amplify levels to those found in aged WT controls, resisted both the adverse effects of age and acute stress on spatial memory. Such memory effects are not a consequence of altered hippocampal MR and GR levels that do not differ between WT and 11β-HSD1^−/−^ mice (Yau et al., 2011).

It is well established that CORT enhances memory consolidation when administered immediately after training on emotionally arousing learning tasks such as inhibitory avoidance, water maze, and object recognition (Okuda et al., 2004, Roozendaal and McGaugh, 1997, Roozendaal et al., 1996). The increase in intrahippocampal CORT levels in freely behaving mice during exploration of the Y-maze are consistent with the anticipated rise in plasma CORT after exposure to a novel environment (Fluttert et al., 2000). Previous studies that gave exogenous CORT at different phases of learning (Conrad et al., 1999, Quervain et al., 1998, Roozendaal, 2002) or varied endogenous CORT levels by adjusting stress intensity during the learning process showed that CORT effects on memory follow an inverted U-shaped dose-response pattern (Salehi et al., 2010). Our findings here, using a direct method of measuring brain CORT levels in vivo simultaneously with behavioral testing in mice, support a biphasic effect of glucocorticoids on memory (Park et al., 2006, Salehi et al., 2010). Thus, the modest increases in endogenous “free” and, hence, biologically active CORT levels in the hippocampus during the acquisition (trial 1) and retrieval (trial 2) phases of the Y-maze task are consistent with predominant MR activation (Yau et al., 2011). This pattern of response was found in young WT controls, in aged WT mice treated with UE2316, and in young and aged 11β-HSD1^−/−^ mice. In contrast, impaired memory was associated with the greater and longer lasting increases in hippocampal CORT levels in aged WT controls and young stressed WT mice supporting predominant GR activation (Yau et al., 2011). Acute stress (tail nick) before retrieval impaired memory in young WT mice consistent with similar findings in rats (de Quervain et al., 1998). The deleterious effects of a rapid rise in hippocampal CORT levels occurring within minutes suggest a nongenomic mechanism possibly via membrane receptors (Karst et al., 2005) with later genomic effects mediated via “classical” intracellular MRs and GRs (Oitzl et al., 2001). Indeed, both rapid and delayed CORT effects seem to be involved in the acquisition, consolidation, and retrieval phases of memory (Groeneweg et al., 2011).

The link between circulating CORT levels and memory impairment appears to involve local production of active CORT because 11β-HSD1^−/−^ mice subjected to acute stress retain the memory of a learned task even when circulating CORT levels are increased. Although aging enhanced the stress-induced increase in intrahippocampal CORT levels in WT mice in line with the previous findings (Tronche et al., 2010), no age effect was observed in 11β-HSD1^−/−^ mice. It is likely that the stress-induced rise in intrahippocampal CORT levels in 11β-HSD1^−/−^ mice, which derives from peripheral-free CORT, is enough to activate MR to maintain spatial memory but is insufficient to activate the GR-dependent impairment of spatial memory (Yau et al., 2011). This concept is supported by observations of the effects of metyrapone that alters the balance of MR and GR ligands by inhibiting glucocorticoid synthesis and promoting the synthesis of a mineralocorticoid, 11-deoxycorticosterone. Metyrapone is also a competitive substrate of 11β-HSD1 (Sampath-Kumar et al., 1997) and has been shown to block the stress-induced increases in intrahippocampal CORT and also the memory-impairing effects of stress (Chauveau et al., 2010, Tronche et al., 2010).

Intrahippocampal CORT levels measured in freely behaving mice under basal conditions before and after Y-maze testing were surprisingly similar between the genotypes, particularly in young mice. Equilibrium between plasma-free CORT and hippocampal intracellular CORT (Qian et al., 2012) appears well maintained when CORT levels are low even in the absence of 11β-HSD1. Consistent with this, measures of ex vivo hippocampal tissue steroids in young 11β-HSD1^−/−^ mice showed only a trend toward lower CORT levels (see Table S1 and Cobice et al., 2013). The direct contribution of 11β-HSD1 to intrahippocampal CORT levels and spatial memory appears most pronounced when plasma CORT levels were markedly elevated as with aging or after acute stress. Both in vivo intrahippocampal CORT (during Y-maze) and ex vivo hippocampal tissue CORT levels were significantly lower in aged 11β-HSD1^−/−^ mice compared with aged WT mice consistent with our previous findings (Sooy et al., 2010, Yau et al., 2011). Increased 11β-HSD1 substrate (11-dehydrocorticosterone) availability through peripheral CORT breakdown by 11β-HSD2 may underlie the rapid increase in intrahippocampal CORT levels during Y-maze exposure and after tail-nick stress. Moreover, glucocorticoids themselves may upregulate transcription of 11β-HSD1 (Sai et al., 2008), thus contributing to the increased intrahippocampal CORT levels. Indeed, stress and aging have previously been shown to increase hippocampal 11β-HSD1 expression (Holmes et al., 2010, Low et al., 1994).

In conclusion, local intracellular regeneration of active glucocorticoids by 11β-HSD1 in the hippocampus is dynamically regulated during learning. This regenerative component is even greater with aging reflecting an associated increase in 11β-HSD1 expression in the hippocampus (Holmes et al., 2010). Our data show that it is this rise in hippocampal CORT levels amplified by 11β-HSD1 activity during the retrieval phase of spatial learning that appears to impair memory in aged WT controls or stressed young WT mice. Whether other brain regions that express 11β-HSD1 are also involved in the memory impairment remains to be determined. Furthermore, the observation that preventing local amplification of CORT in 11β-HSD1^−/−^ mice protects against stress-induced spatial memory impairment has important implications for the development of treatments for age-related memory deficits. They suggest that local acute CORT changes in the hippocampus at the time of learning is in part responsible for glucocorticoid-dependent memory impairments, rather than the chronic cumulative effects of elevated plasma CORT levels on hippocampal morphology and function (Landfield et al., 2007, Lupien et al., 1998, Sapolsky, 1996). Previous studies in rats and humans also indicate that memory deficits observed during periods of chronic elevated glucocorticoids are, in part, readily and acutely reversible by pharmacologic intervention (Coluccia et al., 2008, Wright et al., 2006). Indeed, in the present study, short-term treatment with a selective 11β-HSD1 inhibitor, UE2316, prevented the dynamic amplification of intrahippocampal CORT levels during Y-maze learning and reversed spatial memory impairments in already aged WT mice. Other approaches to reduce CORT actions in brain such as GR antagonists have also been shown to reverse age-related memory deficits (Yau et al., 2011) and restore changes in hippocampal plasticity and neurogenesis after chronic stress or high CORT exposure (Krugers et al., 2006, Zalachoras et al., 2013). However, GR antagonists such as RU486 also interfere with glucocorticoid negative feedback resulting in increased CORT levels (Spiga et al., 2007) and reduced efficacy. RU486 is also a progesterone receptor antagonist. Thus, RU486 may cause side effects from progesterone receptor antagonism and/or peripheral GR activation. 11β-HSD1 inhibitors, in contrast, do not bind to GRs and have shown no significant effect on plasma glucocorticoid levels. Our data also suggest that when selective 11β-HSD1 inhibitors are used to treat chronic age-related memory deficiencies, there may be the added advantage of protection from acute stress-induced memory impairments.

## Disclosure statement

Development of UE2316 was supported by a Wellcome Trust Seeding Drug Discovery Award (to BRW, JRS, and SPW). SPW, BRW, and JRS are inventors on patents owned by the University of Edinburgh that encompass the use of UE compounds for treatment of cognitive dysfunction. The other authors report no financial interests or potential conflicts of interest.
