# Supplementary figures and images for: Intrahippocampal glucocorticoids generated by 11β-HSD1 affect memory in aged mice
Source: Neurobiol Aging. 2015 Jan;36(1):334–43. doi: 10.1016/j.neurobiolaging.2014.07.007 (PMC4706164; doi:10.1016/j.neurobiolaging.2014.07.007)

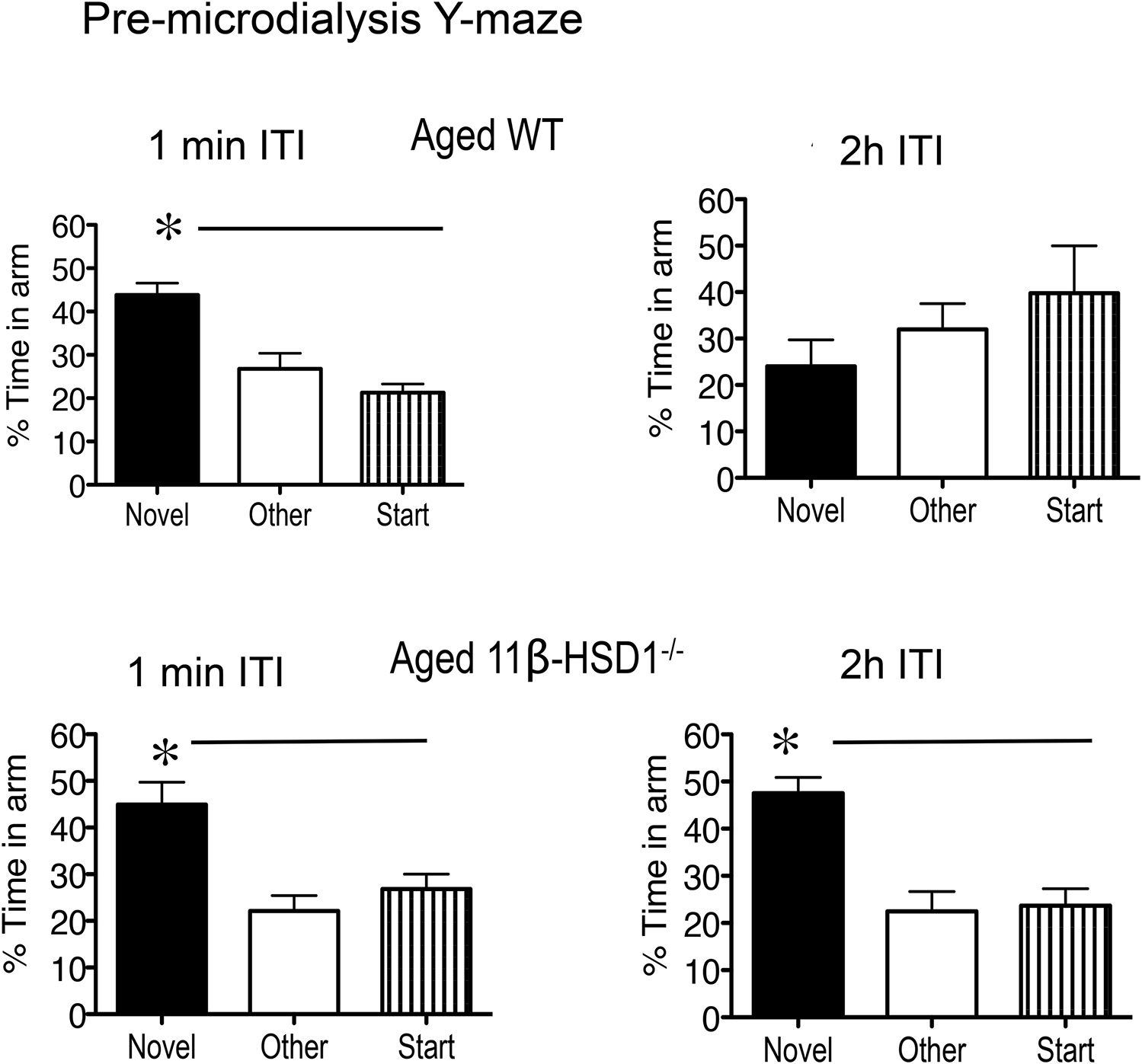

Supplement: Supplementary Fig. S1 [file figs1.jpg]

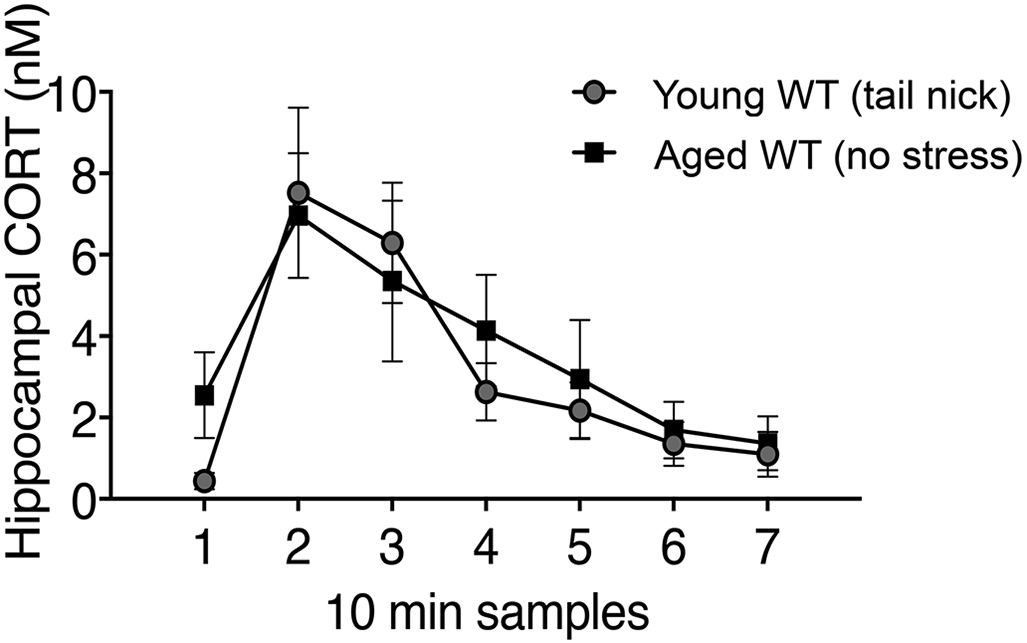

Supplement: Supplementary Fig. S2 [file figs2.jpg]

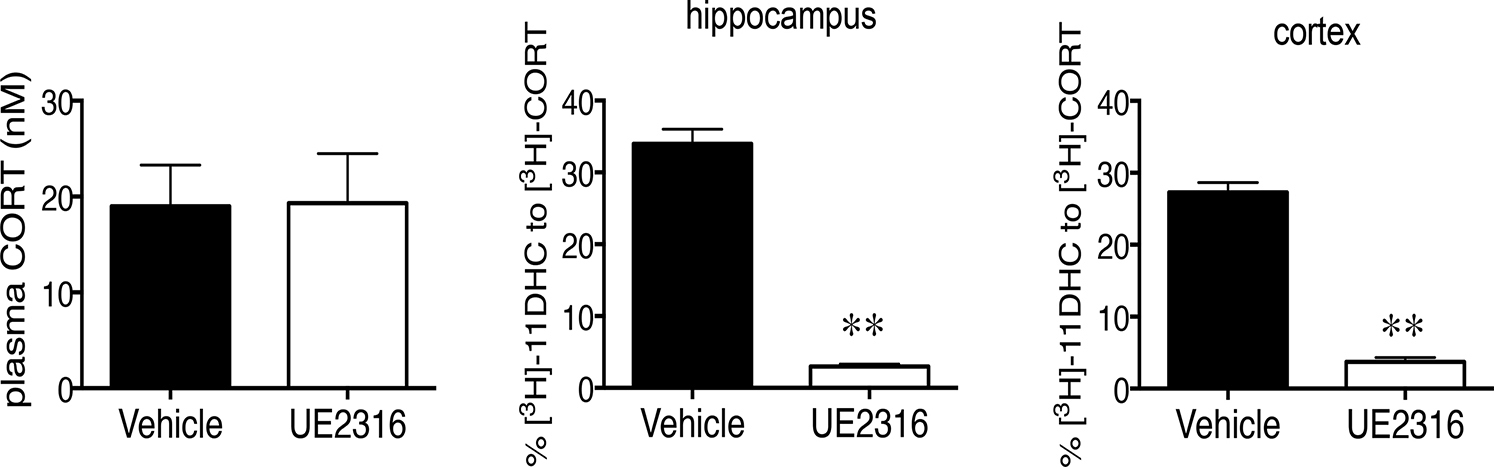

Supplement: Supplementary Fig. S3 [file figs3.jpg]
