# Supplementary material for: Intrahippocampal glucocorticoids generated by 11β-HSD1 affect memory in aged mice
Source: Neurobiol Aging. 2015 Jan;36(1):334–43. doi: 10.1016/j.neurobiolaging.2014.07.007 (PMC4706164; doi:10.1016/j.neurobiolaging.2014.07.007)
Supplement: Supplementary Table 1 [file mmc1.doc]

JLW Yau *et al*

**Table S1 Effect of age and 11β-HSD1 deficiency on basal**

AM plasma and ex-vivo hippocampal CORT levels.

| Genotype | Age (m) | Plasma CORT (nM) | Hippocampal CORT (ng/g) |
| --- | --- | --- | --- |
| Wild type | 6 | 39.0 ± 6.7 | 33.5 ± 3.5 |
|  | 24 | 70.7 ± 6.0** | 45.9 ± 3.5* |
| 11β-HSD1^-/-^ | 6 | 48.3 ± 8.3 | 26.0 ± 3.0 |
|  | 24 | 71.0 ± 9.3** | 29.6 ± 2.5§ |

**P<0.01, *P<0.05 compared to corresponding young mice;

§P<0.01 compared to aged wild type controls. Values are means ± SEM.
