# Supplementary material for: Intrahippocampal glucocorticoids generated by 11β-HSD1 affect memory in aged mice
Source: Neurobiol Aging. 2015 Jan;36(1):334–43. doi: 10.1016/j.neurobiolaging.2014.07.007 (PMC4706164; doi:10.1016/j.neurobiolaging.2014.07.007)
Supplement: Supplementary Figure Legends [file mmc2.docx]

**Figure S1. Aged 11β-HSD1^-/-^ mice resists the spatial memory impairments in aged wild type (WT) mice**. Two weeks prior to commencing the combined *in vivo* microdialysis and Y-maze testing in aged mice, the animals were first tested in the immediate version of the Y-maze (1min inter-trial interval (ITI)) followed 3 days later with the 2h ITI test. This prior testing was carried out to confirm that the microdialysis procedure *per se* had no effect on the cognitive phenotype of the aged mice.

**Figure S2. Tail nick stress-induced increases in intrahippocampal CORT in young wild type (WT) mice comparable to aged WT mice levels.** Intrahippocampal levels in young 6 months old WT controls given tail nick stress immediately before retrieval in 7 consecutive 10 min dialysate samples collected during and after Y-maze (trial 2) entry from figure 3(B) superimposed onto intrahippocampal CORT levels in aged 24-26 months old WT controls during and after Y-maze (trial 1) entry without tail nick stress from figure 4(B).

**Figure S3. Reduced 11β-HSD1 activity in mouse brain following oral UE2316.** UE2316 was first tested in young 6 months old wild type mice to confirm effective inhibition of brain 11β-HSD1. Five days of oral UE2316 treatment (10mg/kg or vehicle twice daily in jelly, n=5/group) did not affect morning plasma CORT levels but potently reduced 11β-HSD1 activity in hippocampus and cortical tissues collected 1h after the final dose of inhibitor. 11β-reductase activity measured in brain tissue homogenates incubated with [^3^H]-11dehydro-CORT. The % conversion to [3H]-CORT was measured as amount of 11β-reductase activity. **P<0.0001 compared to vehicle controls.
